# Revisiting Apathy in Alzheimer's Disease: From Conceptualization to Therapeutic Approaches

**DOI:** 10.1155/2021/6319826

**Published:** 2021-08-03

**Authors:** Antonio L. Teixeira, Mitzi M. Gonzales, Leonardo Cruz de Souza, Sara L. Weisenbach

**Affiliations:** ^1^Neuropsychiatry Program, UT Health Science Center at Houston, USA; ^2^Instituto Ensino e Pesquisa, Santa Casa BH, Belo Horizonte, Brazil; ^3^Glenn Biggs Institute for Alzheimer's & Neurodegenerative Diseases, UT Health Science Center at San Antonio, USA; ^4^Programa de Pós-Graduação em Neurociências, Universidade Federal de Minas Gerais (UFMG), Belo Horizonte, Brazil; ^5^Departamento de Clínica Médica, Faculdade de Medicina, UFMG, Belo Horizonte, Brazil; ^6^Department of Psychiatry & Behavioral Health, Stony Brook University, Stony Brook, New York, USA

## Abstract

Apathy is a neurobehavioral syndrome characterized by impaired motivation for goal-directed behaviors and cognitive activity, alongside blunted affect. Apathy is a common neuropsychiatric syndrome in Alzheimer's disease (AD), with a 5-year prevalence over 70%. Apathy also serves as a prognostic indicator, correlating with the progression of AD. Despite advances in its conceptualization and understanding of its neural basis, there is very limited empirical evidence to support the available strategies for the treatment of apathy in AD. Given its complex pathophysiology, including distinct substrates for different apathy dimensions (affective, cognitive, and behavioral), it is unlikely that a single pharmacological or nonpharmacological strategy will be effective for all cases of apathy in AD. High-quality evidence research is needed to better understand the role of specific strategies aiming at a personalized approach.

## 1. Introduction

Apathy is a neurobehavioral syndrome characterized by reduced or loss of motivation for self-initiated goal-directed behaviors and cognitive activity, alongside blunted affect [1, 2]. Apathy is commonly regarded as the most common neuropsychiatric syndrome in Alzheimer's disease (AD) [3–6]. Its frequency varies according to the population studied: a point prevalence of around 50% in outpatient settings and 35% in community samples of subjects with AD, with a 5-year prevalence over 70% [3–6].

In contrast to other neuropsychiatric syndromes (e.g., anxiety and depression) that might have a fluctuating course, apathy is stable over time, correlating with the progression of AD [7, 8]. Accordingly, apathy has also been found to predict progression from normal cognition to MCI [9, 10] and from MCI to AD dementia [11–13]. Apathy has also been associated with negative outcomes in people with dementia, including greater functional and cognitive impairment, frailty [14], greater caregiver burden [15], increased risk of institutionalization [16], and even higher mortality [17].

Because of its prevalence, status as a prognostic indicator and functional significance of disease, apathy is a relevant target in the management of patients with AD. This manuscript is aimed at scoping the literature about apathy in AD to clarify the concept of apathy alongside its assessment and treatment in patients with AD and to identify knowledge gaps. For this scoping review [18], we searched the pertinent literature on the PubMed database until June 2021, focusing on meta-analyses, systematic reviews, and original studies published in the last five years, also including pivotal studies.

## 2. The Concept and Assessment of Apathy

The contemporary investigation of apathy dates back to the seminal papers in the 1990s by Robert Marin who provided a highly influential definition based on “the loss of motivation not attributed to intellectual impairment, or diminished level of consciousness” [19, 20]. Later, Starkstein et al. proposed the three core features of apathy: diminished motivation, diminished initiative, and blunting of emotions [1]. In 2006, Levy and Dubois [21] also made an influential contribution, defining apathy “as a quantitative reduction of voluntary, goal-directed behaviors.” Accordingly, apathy could be divided into three subtypes: emotional-affective, cognitive, and auto-activation (i.e., lack of spontaneous activation to environmental stimuli).

In 2008, the European Psychiatric Association commissioned a task force led by Robert et al. to develop categorical diagnostic criteria for apathy in AD [22]. The criteria were recently revised, defining apathy within a framework similar to the Diagnostic and Statistical Manual of Mental Disorders (DSM). Accordingly, an individual with AD is diagnosed as apathetic when he/she meets four criteria (A-D) [23]. Criterion A requires a quantitative reduction of goal-directed activity in behavioral/cognitive, emotional, and/or social dimensions compared to his/her previous level of functioning. Criterion B specifies the presence of symptoms in at least two of these three domains for at least four weeks and present most of the time, providing respective examples related to auto-activation (or spontaneous) and response to environmental stimulation. For instance, in the social interaction domain, the patient is less likely to initiate a conversation (impaired auto-activation) or withdraws soon from it (impaired response). Criterion C states that these symptoms cause significant impairment in personal, social, occupational, or other areas of functioning. Finally, criterion D stipulates these symptoms cannot be explained by physical (e.g., blindness or deafness) or motor disabilities, impaired arousal, or the direct effect of medication or drugs. It is worth highlighting that the latter criterion demands the exclusion of a hypoactive or mixed delirium.

Apathy is a transdiagnostic neurobehavioral syndrome present not only in AD but also in other neurodegenerative diseases and psychiatric conditions [2, 24]. In Huntington's disease, for example, apathy can manifest quite early in the course of the disease, even antedating the development of typical motor signs that clinically define the condition [25, 26]. In the behavioral variant of frontotemporal dementia (bvFTD), apathy (or “inertia”) can be an important behavioral feature for the diagnosis in addition to disinhibition, loss of empathy, hyperorality, and stereotyped behavior [27–29]. Apathy is also a central element of the negative syndrome of schizophrenia [30].

As an independent syndrome, apathy is less investigated in the context of mood disorders. This can be explained, at least in part, by the fact that the symptom “lack of motivation” is one of the criteria for the diagnosis of a major depression episode, making it challenging to disentangle the two disorders. In addition, anhedonia, i.e., loss or diminished pleasure in usual activities, which is a core symptom of major depression, also frequently overlaps with apathy—although they likely map onto distinct neural substrates within reward-related circuits [31–33]. Multiple studies have indeed shown a considerable overlap—30-50%—between depression and apathy in patients with AD [34–37]. From a clinical standpoint, the apathy syndrome differentiates from depression as the former does not involve subjective feelings of sadness and negative thoughts.

Rating scales are the most common approach for measuring apathy in AD. Several of the core diagnostic criteria for apathy are integrated in the clinician-rated questionnaire informed by caregivers and patients with AD [38, 39]. Besides generic instruments available to evaluate multiple neuropsychiatric syndromes in AD, with the Neuropsychiatric Inventory (NPI) being the most frequently used in research and clinical settings [40], there are tools specifically developed to assess apathy. The latter group includes the Apathy Evaluation Scale (AES) [19, 41], the Apathy Inventory [42], the Lille Apathy Rating Scale [43, 44], and the Dimensional Apathy Scale [45, 46], among others [39, 47].

The most broadly used measure of apathy is the AES. The AES, originally developed by Marin [19, 41] to assess apathy in people with different neurological conditions including AD, provides a comprehensive assessment of apathy. The AES has 18 specific items to quantify apathy within a scoring range from 18 to 72 with higher scores indicating greater apathy. There are three versions: (1) self-report (AES-S), (2) informant report such as a caregiver (AES-I), or (3) clinician rating (AES-C). The questions are the same with only the pronoun referring to the subject changed. However, self-reported AES have been found less reliable than informant- and clinician-based scores [48], which may be attributable, at least in part, to impaired awareness in AD [49]. Suggested cut-off scores are 36.5 (AES-S), 41.5 (AES-I), and 40.5 (AES-C) [50]. Accordingly, in clinical practice, the assessment of apathy must involve not only the patient but also a relative and/or a caregiver, as patients frequently misjudge their engagement in personal and social activities, and their overall level of interest and motivation. As a word of caution, caregivers can also misinterpret apathy. While professional caregivers may not endorse apathy as a distressing or challenging problem, family caregivers may see apathy as an oppositional or deliberate behavior with enhanced levels of related distress [51].

## 3. Neural Correlates of Apathy

Apathy has been associated with disruption in the prefrontal cortex (PFC) and/or the prefrontal-subcortical circuits in AD [52–54]. Both neuropathological and neuroimaging studies have specifically implicated the anterior cingulate cortex (ACC) and orbitofrontal cortex (OFC) in apathy associated with AD [54, 55]. Apathy in AD correlates with a greater neurofibrillary tangle burden in ACC [56]. Structural neuroimaging studies have shown reduced volumes in the ACC and prefrontal cortex, especially OFC, in AD patients with apathy relative to individuals without apathy [57]. There has also been evidence of widespread microstructural white matter abnormalities in AD patients with apathy, suggesting that apathy may arise through disconnection between these and other brain regions [58]. From a theoretical perspective, one may hypothesize that apathy may result from functional and/or structural damage of basal ganglia-thalamo-cortical circuits. More specifically, the cortico-subcortical loop involving anterior cingulate, ventral striatum, and pallidum has been implicated in apathy [59, 60]. It is worth mentioning that basal ganglia-thalamo-cortical circuits are affected across different neuropsychiatric syndromes in AD. However, there are some specific patterns of brain lesions, such as the anterior cingulate-subcortical circuit is specifically related to apathy in AD, the frontal-limbic circuit is related to depression, and the amygdala circuit is related to anxiety [61].

Different functional imaging modalities, including functional magnetic resonance imaging (fMRI), positron emission tomography (PET), and single photon emission computed tomography (SPECT), have demonstrated association between apathy and, respectively, altered functional networks [62, 63], hypometabolism [64–66], and hypoperfusion [67–69] in the ACC and OFC areas of AD patients. For example, a SPECT study found decreased perfusion in left ACC and right OFC in AD patients with apathy relative to those without apathy [69], while a PET study observed hypometabolism in the bilateral ACC and medial OFC [64].

As mentioned above, Levy and Dubois categorized apathy in three different subtypes—emotional-affective, cognitive, and auto-activation. Each subtype is presumed to be governed by distinct neural circuitry. The emotional-affective subtype has been associated with lesion/dysfunction in OFC and related limbic territory (e.g., ventral striatum and ventral pallidum). The cognitive subtype has implicated dorsolateral PFC and caudate nucleus lesion/dysfunction. The auto-activation subtype has been linked with the associative and limbic areas of globus pallidus. While this is a compelling model, empirical validation is unfortunately lacking in AD.

Apathy is a frequent neurobehavioral syndrome in other neurodegenerative diseases, such as behavioral variant frontotemporal dementia (bvFTD) and Parkinson's disease. To a certain extent, all these neurodegenerative diseases share overlapping neural correlates of apathy [28, 70]. Nevertheless, recent neuroimaging investigations have contributed to refine the clinical phenomenology of apathy, by demonstrating specific neural underpinnings across AD and other neurodegenerative dementias. For instance, in a behavioral and neuroimaging (PET-FDG) comparison between AD and bvFTD, Fernandez-Matarrubia et al. found that bvFTD patients have more deficits in emotional apathy and self-awareness, suggesting that apathy in AD is less “affective” than in bvFTD [71]. Of note, each group of patients had different patterns of correlations between apathy scores and brain metabolism: while apathy correlated with right anterior cingulate in AD, bvFTD patients had more widespread correlations in PFC, including lateral orbitofrontal and anterior insular cortices. These findings were confirmed by an independent group who found that apathy is common in both AD and bvFTD, but with distinct phenomenological manifestations, with AD patients exhibiting only cognitive apathy and bvFTD presenting both affective and cognitive apathy [72]. While cognitive apathy correlated with the dorsomedial PFC, affective apathy correlated with the ventral PFC [72].

It is worth highlighting that neural mechanisms alone do not completely account for apathy, and other determinants include individual, caregiver, and environmental factors [73]. Current conceptual models of apathy acknowledge that the syndrome is the result of a combination of direct (i.e., degeneration-induced neural circuit disruptions) and indirect (e.g., presence of other symptoms and need of caregiver) effects of AD alongside environmental and other factors [52, 73, 74].

## 4. Therapeutics: Pharmacological and Nonpharmacological Interventions

There is limited empirical evidence to support the available strategies for the treatment of apathy in AD [75–79]. These strategies can be grouped in three categories: pharmacological, neuromodulation, and behavioral.

Standard pharmacological approach for apathy in AD has relied on optimized use of the Food and Drug Administration-approved drugs for AD, i.e., cholinesterase inhibitors (e.g., donepezil and rivastigmine) and memantine [74]. While older studies reported evidence of mild effectiveness of cholinesterase inhibitors on apathy, this was not replicated in more recent investigations [75, 77]. Despite that, some authors argue that cholinesterase inhibitors may be the best pharmacological strategy for the treatment of apathy in AD [79].

As apathy has been conceptualized within the disorders of motivation and reward [31–33, 70], where dopaminergic circuits play a pivotal role, pharmacological approaches stimulating dopamine signaling have been used in the treatment of apathy in AD. The stimulant methylphenidate was shown to be effective in reducing apathy in AD in open studies and two double-blind randomized controlled trials [40, 80, 81]. However, the use of methylphenidate was associated not only with reduction in apathy but also with greater anxiety and weight loss [40]. Another concern with the use of stimulants is their potential cardiovascular effects, a fact particularly relevant in older adults with multiple medical comorbidities [82]. Modafinil, a waking-promoting agent, was not effective in reducing apathy in patients with AD or caregiver burden [83]. Dopamine agonists, such as rotigotine, have been investigated for the treatment of apathy in different neuropsychiatric conditions, especially Parkinson's disease [84, 85]. In AD, a recent randomized, double-blind, placebo-controlled trial did not show any significant effect of rotigotine 4 mg transdermal patch on global cognition and NPI scores, but improvement of frontal lobe cognitive measures and functioning in activities of daily living [85]. Therefore, a more in-depth appraisal of the role of dopamine agonists in the treatment of apathy in AD is warranted.

Regarding antidepressants, especially the selective serotonin reuptake inhibitors (SSRIs), while they can be useful to treat comorbid depressive symptoms, there is no evidence to support their use for apathy in AD [75]. Actually, there are reports of worsening apathy in patients with neurodegenerative diseases taking SSRIs [86].

Noninvasive brain stimulation techniques, such as repetitive transcranial magnetic stimulation (rTMS) and transcranial direct current stimulation (tDCS), have emerged as promising therapeutic tools for AD [87]. Transcranial direct current stimulation (tDCS) is a relatively novel nonpharmacological method of neuromodulation that has been evaluated in several neuropsychiatric conditions, showing positive results in depression and negative symptoms (including apathy) of schizophrenia [88, 89]. In AD, a few controlled studies have been conducted to evaluate the role of tDCS on cognitive functioning. A systematic review and meta-analysis of these studies found that tDCS improved cognitive function in mild to moderate AD, but the stimulation parameters (multiple sites; single vs. repeated; lower vs. higher current) were very different among studies, not allowing definite conclusions [90]. Of note, Suemoto et al. studied 40 patients with AD who were randomized to receive either anodal tDCS (2 mA constant current for 20 minutes) or sham-tDCS over the left dorsolateral PFC for six sessions during two weeks [91]. While tDCS was safe in this population, there was no evidence of efficacy of tDCS on apathy nor on the other neuropsychiatric symptoms assessed. The lack of efficacy was attributed to several factors, including the low number of sessions and the short period of intervention [91]. A similar scenario is observed for rTMS. While the effectiveness of rTMS for AD is still unclear, at least in part due to methodological issues (low statistical power and heterogeneity of studies) [92], a recent systematic review and meta-analysis of the available trials showed medium-to-large effect size of rTMS in the improvement of cognitive functions [93]. Fewer studies evaluated the role of rTMS in neuropsychiatric symptoms in AD. When neuropsychiatric symptoms have been examined, they are usually assessed as a secondary outcome and without specifying the symptom. There is preliminary evidence to suggest that rTMS might be effective for attenuating their severity [94]. A very recent preliminary study of apathy in AD found that stimulation to the left dorsolateral prefrontal cortex was associated with greater improvement in AES-C relative to sham treatment [95].

Studies investigating behavioral strategies for apathy in AD, such as music, art therapy, and exercise, have shown modest effects, mainly in subjects in the early stages of dementia [96–98]. However, these studies were very heterogeneous from a methodological standpoint, sometimes lacking conceptual clarity and specificity [97].

## 5. Future Directions

Despite advances in the conceptualization and understanding of the pathological basis of apathy in AD, there are several gaps to be addressed. The availability of an internationally recognized criteria for apathy diagnosis [23] provides a framework to evaluate the validity and applicability of the construct into research and clinical settings. However, apathy subtypes, including their interaction with other cognitive and behavioral domains, remain to be thoroughly investigated. In this context, emerging technologies (e.g., wearable devices) might help a better quantification of different components of apathy syndrome [99]. A related issue is to evaluate whether subtypes correlate with discrete neural circuitry dysfunction in AD, as previously proposed [21, 73], which may enable the development of more targeted interventions.

Robustly effective pharmacological approaches remain to be developed [75–77]. Accordingly, well-designed clinical trials controlling for potential confounders (e.g., severity of dementia, concurrent depression, medical comorbidities, and polypharmacy) with apathy as the primary outcome must be carried out [100]. Behavioral interventions for apathy, such as Behavioral Activation Therapy, also deserve attention for future clinical trials. Furthermore, a better understanding of the multiple determinants of apathy is critical for effective treatment. Given its complex pathophysiology, including distinct substrates for different apathy dimensions (e.g., affective, cognitive, and behavioral), it is unlikely that a single pharmacological or nonpharmacological strategy will be effective for all cases of apathy in AD, but personalizing treatment is still elusive. As for the assessment of apathy, emerging technologies (e.g., tablet-based and exergaming) can play a role in its management, supporting tailored activities for patients [101, 102]. In some cases, a caregiver-centered approach might lead to better results than a patient-centered one. For that, high-quality evidence research is needed to better understand the role of caregiver and environmental factors on apathy development and exacerbation and how to properly intervene on these factors [73]. In addition, the synergistic effect of pharmacological and nonpharmacological strategies and/or stepped approaches starting with behavioral measures remains to be explored.

From a neurobiological perspective, the role of processes beyond the effects of neurodegeneration on neurotransmitters and/or neural circuits must be investigated. For example, apathy has been associated with increased circulating levels of inflammatory mediators in older adults and patients with AD [103–105]. This might open new venues for therapeutic intervention, in this instance, based on anti-inflammatory strategies, as it has been proposed for mood disorders and other neurodegenerative diseases [106–108]. Neurophysiological biomarkers that can be assessed with noninvasive brain stimulation techniques also used for therapeutics should be explored in relation to apathy, its dimensions, and other neuropsychiatric symptoms in AD [109]. Finally, as a transdiagnostic neurobehavioral syndrome, the understanding of apathy in AD might benefit from the study of apathy in other neurodegenerative diseases and psychiatric conditions and vice-versa. With further research, clinicians and researchers may be able to effectively mitigate apathy in individuals with AD, offering improved quality of life for affected individuals and their families.
